# Population awareness of diabetic eye disease and age related macular degeneration in Nepal: the Bhaktapur Retina Study

**DOI:** 10.1186/s12886-015-0175-z

**Published:** 2015-12-29

**Authors:** Raba Thapa, Sanyam Bajimaya, Govinda Paudyal, Shankar Khanal, Stevie Tan, Suman S. Thapa, Ger van Rens

**Affiliations:** Tilganga Institute of Ophthalmology, P O Box: 561, Kathmandu, Nepal; Central Department of Statistics, Tribhuvan University, Kirtipur, Nepal; Vrije University Medical Center, Amsterdam, The Netherlands

**Keywords:** Awareness, Age-related macular degeneration, Diabetic retinopathy, Nepal

## Abstract

**Background:**

Diabetic retinopathy (DR) and age-related macular degeneration (AMD) are among the leading causes of visual impairment and blindness in developing countries. This study aims to explore the awareness of these retinal diseases in Nepal.

**Method:**

A population based cross-sectional study conducted among individuals 60 years and older from the Bhaktapur district of Nepal. One thousand consecutive subjects were enrolled and subjected to a structured questionnaire.

**Result:**

Subject age ranged from 60 to 93 years with a meanof 69.5 years ± 7.1(S.D.). Males and females comprised 45.1 and 55.9 % of the population, respectively. The majority was illiterate (78.2 %), and agriculture was the predominant occupation (79.8 %). 12.1 % were aware of the effect of diabetes on the eye, and among them, 99 % were aware that diabetes was a blinding disease caused by DR.11.5 % of the subjects were aware of DR, and 10.1 % were aware that subjects with diabetes should undergo periodic eye examinations. Only 7.6 % of subjects were aware of AMD.7.5 and 7.4 % were aware about its aggravation with smoking and sunlight exposure, respectively. Younger age group, males, literates, service holders, best corrected visual acuity >0.3 LogMAR, were each significantly associated with an increase in awareness of diabetic retinopathy. Smokers and those with agricultural occupations were less aware regarding AMD. Those with diabetes,with or without DRwere significantly more aware than those not having the disease.

**Conclusion:**

Among the Bhaktapur population, awareness of DR and AMD was only 11.5 and7.6 % respectively. Older age groups, females, illiterates, farmers, and those with poor visual acuity were less aware of these blinding diseases. We recommend community-based eye health education programs targeted at raising awareness of these diseases and preventive measures.

## Background

Diabetic retinopathy (DR) and age related macular degeneration (AMD) are the major causes of visual impairment and blindness worldwide [1–5]. In the developing world, diabetes is now recognized as a major public health conditions due to changes in lifestyle [6]. Diabetic retinopathy is the fifth leading cause of global blindness and the most important cause of blindness among working age individuals. The increased incidence of diabetes has led DR to be an important cause of blindness in the developing world [7]. AMD, the third leading cause of global blindness and the most common cause of irreversible blindness among the elderly in the developed world, is also a leading cause of blindness in the developing countries [8]. A study in Nepal reported that AMD was a major cause of blindness with a prevalence of blindness 8.7 % [9]. The prevalence increased with life expectancy, and lack of awareness could be a contributing factor [8, 9].

Blindness from DR and AMD is often preventable since progression is treatable if the disorder is detected early [10]. Raising awareness of modifiable risk factors not only would help reduce the onset of disease and its progression but also would encourage people to seek regular eye examination for early detection [11]. Patients usually present late in the course of the disease, mainly due to lack of awareness [12]. Limited access and availability of retinal services in Nepal also add to the problem because of the limited numbers of eye hospitals and eye care providers [12, 13].

This is the first population-based study to explore awareness of major retinal diseases such as DR and AMD in Nepal.

## Methods

The Bhaktapur Retina Study is a population-based, cross-sectional study to estimate the prevalence of vitreo-retinal diseases among subjects 60 years and above residing in the Bhaktapur district of Nepal. This is the second survey on the same cohort that was enrolled for the Bhaktapur Glaucoma Study (BGS) conducted between 2007 and 2010. Details on demographics of the study group have been described elsewhere [14]. In brief, study participants were enrolled from 30 clusters of the district. A sample size of 2100 was calculated after assuming 7 % prevalence for vitreo-retinal disorders in individuals 60 years and older, a relative precision of 25 %, 85 % compliance, and a design effect of 2. The 7 % prevalence of vitreo-retinal disorder was derived from the occurrence of retinal disorders in the BGS [4]. All subjects attended the community eye centre in the Bhaktapur district and underwent an ocular examination. 1000 consecutive subjects of the total of 2100 attending from August 2013 to September 2014 were enrolled in this study.

A structured questionnaire was developed to assess the awareness of diabetic ocular problems and AMD. The questionnaires on diabetic ocular problems focusedon the effect of diabetes on eyesight, awareness of diabetic retinopathy,and the need to visit an eye specialist. If aware of DR, the source of awareness was also asked. The questionnaire for AMD focused on awareness of AMD, association with smoking, sunlight exposure and the protective effect of consumption of green leafy vegetables, fish, and antioxidant vitamins.

The questionnaire was developed specifically for this study to assess awareness of these two blinding diseases. A total of four questions focused on diabetic ocular problems, and five questions were directed toward AMD and DR. The questionnaires were pretested before final enrollment in the studyand were administered prior to the eye examination. Mid-level ophthalmic personnel were involved in the interview, and 50 cases were pre-tested. No respondents reported difficulties in answering the questionnaire. .

When the subjects were able to read and write in the national Nepali language, they were categorized as literate as defined by the Government of Nepal. The predominant profession was considered as the occupation. The best corrected visual acuity (BCVA) was assessed using the logarithm of minimum angle of resolution (logMAR) with tumbling E charts placed at 4 m. All patients underwent a detailed history, anterior segment and dilated fundus examination including measurement of intraocular pressure. Two retina specialists performed standardized eye examinationson the patients. A total of five fundus photographs were taken of each eye after mydriasis using a Canon digital fundus camera by a trained mid-level ophthalmic technician who had been government certified course to provide primary eye care in ophthalmology.

The blood pressure and random blood sugar were recorded. The diagnosis of diabetes mellitus was based on either the use of diabetic medications or a random blood sugar level of 200 mg/dl or greater. Glycosylated hemoglobin was not measured in this series.

Diabetic retinopathy was graded using Early Treatment Diabetic Retinopathy Study (ETDRS) criteria [10]. Likewise, AMD was categorized according to the international classification developed by the International ARM Epidemiological Study Group [15].

The study was approved by the Institutional Review Board and Ethics Committee of Tilganga Institute of Ophthalmology (TIO) and conducted in accordance with the Declaration of Helsinki. Informed consent was written in the vernacular and was read out for those unable to read. Subjects were asked to sign the consent form, and thumb impressions were taken forthose unable to signprior to enrollment in the study.

### Statistical analysis

Descriptive statistical measures such as mean ± S. Dand percentages were used to summarize continuous variables and categorical variables, respectively. The association between awareness and potentially predictive factors were assessed using aunivariate logistic regression followed by a multiple logistic regression analysis. Those variables which were found significant in a univariate analysis were considered as candidate variables for multiple logistic regressions. We have carried out univariate and multivariate analysis only for awareness of diabetic retinopathy and age-related macular degeneration. For the rest of the questions on awareness, we have reported descriptive analysis only.

The final set of significant factors associated with awareness was identified through the use of a forward stepwise selection procedure with entry probability of 0.05 and removal probability of 0.10. All statistical analyses were performed using STATA 9.0 (StataCorp LD, College Station, Texas, USA). Results were considered statistically significant at 5 % level of significance.

## Results

### Demographic information

**Fig. 1:**
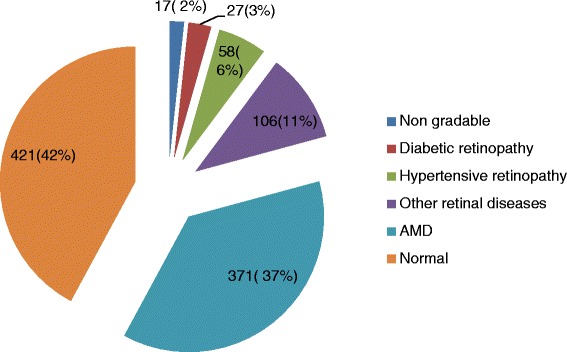
Prevalence of retinal disease among the study subjects (*n* = 1000)

Information regarding awareness was obtained in 97 % of DR cases and 93 % of AMD cases. Ages ranged from 60 to 93 years, with an average age of 69.5 years ± 7.1 S.D. More than half of the cases belonged to the 60–69 year age group. Females (55.9 %) were significantly higher (*p* = 0.012) as compared to males (45.1 %). Due to higher life expectancy in the females, we had more females in the study. Among the total subjects, 768 (76.8 %) were illiterate. Agriculture was the predominant occupation in 799 subjects (79.8 %) followed by housewives in 81 subjects (8.1 %),service holders in 58 subjects (5.8 %) and others in 62 subjects (6.2 %)(Table 1). Diabetes mellitus was found in 85 cases (8.5 %). Duration of diabetes ranged from 2 days to 25 years with an average duration of 6.8 years ± 5.5S.D. Almost two-thirds presented with a history of diabetes duration less than 10 years. Among the total enrolled cases, only 42 % had normal retinal findings, whereas 2 % had non-gradable fundus findings due to hazy media because of cataract. AMD was the most common retinal disease, which was found in 371 subjects (37 %). Diabetic retinopathy comprised of 27 cases (3 %), and hypertensive retinopathy was encountered in 58 cases (6 %). The rest of the106 cases (11 %) had other retinal problems (Fig. 1).

**Table 1:** Demographic profile of enrolled subjects in the study

Characteristics		Number (%)
Age group (years)	60–69 years	518(51.8)
	70–79 years	373(37.3)
	≥80 years	109(10.9)
Gender	Male	451(45.1)
	Females	549(54.9)
Literacy	Illiterate	768(76.8)
	Literate	232(23.2)
Occupation	Agriculture	799(79.9)
	Housewife	81(8.1)
	Service holders	58(5.8)
	Others	62(6.2)

### Awareness of diabetic ocular problems and AMD

Among the enrolled cases,only 10 to 12% of the population was aware of diabetic consequences for the eyes. Among them, 99 subjects (12.2 %) were aware that diabetes mellitus caused damage to the eyes, 112 subjects (11.5 %) had awareness regarding potential decrease in vision, and 99 subjects (10.2 %) were aware of diabetic retinopathy and the need for diabetics to visit their eye specialists regularly (Table 2).

**Table 2:** Awareness of diabetic ocular problems and age related macular degeneration

		Yes (%)	No (%)	Total
Diabetes mellitus	Does diabetes mellitus damage eyes?	118(12.2)	852(87.8)	970
	Does diabetes mellitus impair eye sight?	112(11.5)	858(88.5)	970
	Are you aware of diabetic retinopathy?	99(10.2)	871(89.8)	970
	Should a person with diabetes visit an eye specialist?	99(10.2)	871(89.8)	970
Age-related macular degeneration	Are you aware of AMD?	71(7.6)	858(92.4)	929
	Do you know smoking aggravates AMD?	70(7.5)	859(92.5)	929
	Do you know sunlight exposure aggravates AMD?	69(7.4)	860(92.6)	929
	Do you know green vegetables and fish protect against AMD?	94(10.1)	835(89.9)	929
	Do you know that certain vitamin help slow AMD progression?	89(9.6)	840(90.4)	929

The source of awareness of DR among these 99 diabetic cases who were aware of DR wasfrom doctors including ophthalmologists in42 cases (42.4 %), family members in 24 cases (24.2 %), friends in 9 cases (9.1 %), magazines in 6 cases (6.1 %), and other sources in 18 cases (18.2 %). Similarly, only 7 to 10% were aware of AMD and its associations (Table 2).

### Factors associated with awareness of diabetic retinopathy

The association of awareness of DR and AMD was assessed using logistic regression. Univariate logistic regression analysis showed that younger age, male gender, literates, service holders, BCVA ≥ 0.3 Log MAR, diabetics, longer duration of diabetes,and the presence of diabetic retinopathy each was significantly associated with higher awareness of DR as shown in Table 3. Multiple logistic regressions also showed that the awareness of DR was significantly higher among literates compared to illiterates and between subjects having underlying diabetes mellitus compared to those not having diabetes (Table 5).

**Table 3:** Association of awareness of diabetic retinopathy in the population: Univariate logistic regression

	No (%)	Yes (%)	Total	Odds Ratio	*P* - value	95 % C. I.
Age (Years)						
60–69	435(86.8)	66(13.2)	501	1.00		
70–79	338(93.1)	25(6.9)	363	0.48	0.003	0.3–0.8
≥ 80	98(92.4)	8(7.5)	106	0.53	0.113	0.2–1.1
Sex:						
Male	382(87.4)	55(12.6)	437	1.00		
Female	489(91.7)	44(8.3)	533	0.62	0.028	0.4–0.9
Occupation:						
Agriculture	709(91.3)	67(8.7)	776	1.00		
Service holders	39(72.2)	15(27.8)	54	4.07	<0.001	2.1–7.7
Housewives	71(87.6)	10(12.3)	81	1.49	0.269	0.7–3.0
Others	52(88.1)	7(11.9)	59	1.42	0.402	0.6–3.2
Education:						
Illiterate	690(92.2)	58(7.7)	748	1.00		
Literate	181(81.5)	41(18.5)	222	2.69	<0.001	1.7–4.1
BCVA(Log MAR)						
≥ 0.3	442(86.8)	67(13.2)	509	1.00		
< 0.3	429(93.1)	32(6.9)	461	0.49	0.001	0.3–0.8
Smoking						
No smoking	256(91.1)	25(8.9)	281	1.00		
Present smoker	296(88.9)	37(11.1)	333	1.28	0.365	0.7–2.2
Past smoker	319(89.6)	37(10.4)	356	1.18	0.323	0.7–2.0
Diagnosis of diabetes						
No	820(92.6)	65(7.3)	885	1.00		
Yes	51(60.0)	34(40.0)	85	8.41	<0.001	5.1–13.9
Duration of diabetes (yrs)						
0–4	25(75.7)	8(24.3)	33	1.00		
5–9	15(57.7)	11(42.3)	26	2.29	0.144	0.8–6.9
≥ 10	11(42.3)	15(57.7)	26	4.26	0.011	1.4–12.9
Diabetic retinopathy						
No	853(90.5)	90(9.5)	943	1.00		
Yes	18(66.7)	9(33.3)	27	4.73	<0.001	2.1–10.8

*CI* Confidence interval, *BCVA* Best corrected visual acuity, *AMD* Age related macular degeneration

Among the 27 cases with DR, only 2 cases had prior intervention with laser therapy or anti vascular endothelial growth factor (VEGF). 18 cases of the 27 cases with DR were unaware of DR. Sight threatening retinopathy with clinically significant macular edema (CSME) and proliferative diabetic retinopathy (PDR) was found in 7 cases of the 27 cases with DR. Among these 7 sight threatening cases, five cases were aware of DR. (Table 3).

### Factors associated with awareness of age-related macular degeneration

Univariate logistic regression analysis showed that service holders were significantly more aware of AMD relative to those involved in agriculture work. Awareness was less among the elderly people of the 70–79 years age group, female gender, illiterates, smokers and those with BCVA ≥ 0.3LogMAR as shown in Table 4.

**Table 4:** Association of awareness of age-related macular degeneration in the population: Univariate logistic regression

Characters	No (%)	Yes (%)	Total	Odds Ratio	*P* -value	95 % C I
Age (Years)						
60–69	434(91.2)	42(8.8)	476	1.00		
70–79	324(92.8)	25(7.2)	349	0.79	0.389	0.5–1.3
≥ 80	100(96.2)	4(3.8)	104	0.41	0.099	0.1–1.2
Sex						
Male	378(90.9)	38(9.1)	416	1.00		
Female	480(93.6)	33(6.4)	513	0.68	0.125	0.4–1.1
Occupations						
Agriculture	681(93.2)	50(6.8)	731	1.00		
Service holders	49(84.5)	9(15.5)	58	2.49	0.020	1.1–5.4
Housewives	75(94.9)	4(5.1)	79	0.72	0.544	0.2–2.0
Others	53(86.9)	8(13.1)	61	2.04	0.078	0.9–4.5
Education						
Illiterate	654(93.3)	47(6.7)	701	1.00		
Literate	204(89.5)	24(10.5)	228	1.63	0.061	0.9–2.7
BCVA(Log MAR)						
≥ 0.3	470(91.4)	44(8.6)	514	1.00		
< 0.3	388(93.5)	27(6.5)	415	0.75	0.241	0.4–1.2
Smoking						
No smoking	234(91.4)	22(8.6)	256	1.00		
Present smoker	312(93.1)	23(6.9)	335	0.78	0.430	0.4–1.4
Past smoker	312(92.3)	26(7.7)	338	0.88	0.690	0.5–1.6
AMD cases						
No	541(91.4)	51(8.6)	592	1.00		
Yes	317(94.1)	20(5.9)	337	0.67	0.142	0.4–1.1

*CI* Confidence interval, *BCVA* Best corrected visual acuity, *AMD* Age-related macular degeneration

Awareness of AMD was considerably higher among service holders in comparison to those with agricultural occupations as depicted by the multiple logistic regression analysis (Table 5).

**Table 5:** Multiple logistic regression analysis for awareness of diabetic retinopathy and age-related macular degeneration

	Characters	OR	*P*- value	95 % C.I.
Awareness of Diabetic retinopathy	Age (years)			
	60–69	1.00		
	70–79	0.56	0.025	0.3–0.9
	≥ 80	0.80	0.602	0.3–1.7
	Education			
	Illiterate	1.00		
	Literate	2.38	<0.001	1.5–3.7
	Diagnosis of diabetes			
	No	1.00		
	Yes	7.83	<0.001	4.6–13.1
Awareness of AMD	Occupations			
	Agriculture	1.00		
	Service holders	2.49	0.020	1.1–5.4
	Housewives	0.72	0.544	0.2–2.1
	Others	2.04	0.078	0.9–4.5

*CI* Confidence interval, *AMD* Age related macular degeneration

Out of 337 AMD cases, only 20 subjects were aware of AMD (Table 3). None of them had undergone any specific treatment for AMD. Likewise, among the total 337 AMD cases, 254 cases were smokers.

## Discussion

This is the first population-based study to explore the awareness of DR and AMD in the elderly people of Nepal. Bhaktapur district is situated approximately 15 km away from the capital city, Kathmandu in the mid-mountain region of Nepal. It is divided into two municipalities and 16 village development committees (VDC). Municipalities are taken as urban and VDC’s as rural areas of the district.

Retinal disorders arethe third leading cause of blindness in Nepal, right behind cataract and its iatrogenic sequelae [16]. In the recent Bhaktapur Glaucoma Studythat used the same cohort as our current study, retinal diseases were found to be themost common cause of visual impairment and blindness [17]. A report from the BGS showed that AMDwas the most common retinal disorder followed by DR and retinal vein occlusion among the age group 40 years and above [4]. In our current study,AMD was again identified as the most common retinal disease among the age group 60 years and above.

The awareness of DR among diabetics in our population was only 40 %. Awareness of DR among diabetic patients in another population-based study conducted in an urban area of Nepal was 50 %, which was higher than our study [13]. This difference could have been due to a differencein study age group, which was more than 60 years of age in the present study and above 40 yearsin the urban study. The elderly age group and those residing in rural areas of the district wereless aware of the condition probably due to the limited access to information and low rate of literacy. The overall rate of awareness of DR in our study (11.5 %) was even lower compared to studies conducted in our neighboring country India where the rates of awareness ranged from 19 to 37 % [11, 18, 19]. This could be explained by poorer economic conditions in Nepal when compared to India.

Among the 27 cases with DR, 71 % of the cases that underwent treatment or who suffered from sight threatening conditions were aware of the condition

Doctors and family members were the predominant source for awareness of DR in our study, similar to studies conducted in India [11, 20]. Studies conducted among diabetics attending a hospital have almost always shown a higher awareness (67–87 %) of DR in Nepal and other countries [12, 21–24]. A more comprehensive health care strategy, including timely referral to an ophthalmologist by physicians and other eye care providers, could potentially lead to a higher rate of awareness. Older persons, females, illiterates, farmers, and BCVA < 0.3 Log MAR were significantly less aware about DR in our study, indicating that these groups need to be targeted more specifically.

In our series, only 7.6 % of subjects were aware of AMD. The availability of effective treatment in the modern era combined with the possibility of prompt detection of sight threatening maculopathy for wet AMD has a great potential for reducing the risk of blindness [25]. Remarkably, in a developed country like Australia where awareness of cataract (98 %) and glaucoma (93 %) are high, the awareness of AMD was only slightly higher (20 %) than inour study population [26]. Similarly, a study in Hong Kong revealed awareness of AMD in fewer than 1 % of subjects [27]. Therefore, it appears that the awareness of AMD is universally low and seems independent of the level of awareness of other eye diseases.

Our results show that service holders were remarkably more aware than those involved in agricultural work. Literates had more awareness on AMDthan illiterates. Awareness was considerably lower among the elderly people, females, present and past smokers, and subjects with poor BCVA (<0.3LogMAR). Higher awareness of AMD among the literates and prestigious serviceholders was also found in other large studies in Australia [26, 28]. In one hospital based seriesamong subjects with AMD, 53 % of participants were aware of the importance of antioxidants, and among the 38 % who were using antioxidant vitamins, only 1 % were taking the correct dosage [29]. Cost was the most important factor for not taking the supplementation. Similarly, more than one third of those with advanced AMD were not taking the correct antioxidants in a study conducted in USA [30]. This reflects that the awareness and knowledge regarding the beneficial use of correct vitamin supplementation still needs improvement in developed countries as well. Likewise, awareness regarding the importance of diet, smoking and sunlight exposure is poor around the world.

In the United Kingdom, only 55 % of subjects with AMD were aware that diet was important for eye health and 63 % felt they were not getting enough information about AMD [31]. A hospital-based study in the USA reported less knowledge regarding smoking as a risk factor [32]. Smoking and sunlight exposure are significant risk factors for AMD in studies conducted in Nepal and elsewhere [33–37]. In our series, small percentages (6 %) of those with AMD were aware of their underlying disease, and none had undergone treatment. The majority of the AMD patients were smokers (75.37 %). Since smoking is a risk factor for AMD, awareness regarding cessation of smoking in this population has to be stressed more vigorously.

The low awareness of AMD in smokers and agricultural occupation groupsin our series is of serious public health concern. Improving awareness could potentially reduce the prevalence and progression of AMD in our population.

A survey in the USA showed that 73 % of subjects with DR and 84 % of subjects with AMD were unaware of their diseases [38]. Awareness could motivate people to undergo routine eye check-ups, especially in high risk groups.

Despite the low prevalence of diabetes in developing countries, the rate of blindness from DR is high compared to developed countries. Early detection and early treatment of DR in developed countries could contribute to this disparity [6, 7, 39].

We recommend specifically targeted eye health education programs in the community for preventing blindness from these retinal diseases. Improving literacy seems essential to raising awareness. Furthermore,raising awareness regarding the modifiable risk factors for AMD and DR in younger age groups will help prevent future blindness.

## Conclusion

The overall awareness of vision threatening eye conditions and preventive measures is low in the Bhaktapur district of Nepal. Older persons, females, illiterates, farmers, and subjects with poor visual acuity are particularly unaware of blinding retinal conditions.

These findings highlight the need for comprehensive awareness campaigns. These campaigns are necessary to promote increased awareness in a community by involving people from various walks of life in collaboration with community eye centers and eye hospitals. Improving awareness will help in early detection of diseases and reduction in visual impairment and blindness. We recommend follow up studies of awareness campaigns in the future.

## References

[1] Resnikoff S, Pascolini D, Etya’ale D, Kocur I, Pararajasegaram R, Pokharel GP (2004). Global data on visual impairment in the year 2002. Bull World Health Organ.

[2] Klein R, Klein BEK, Linton KLP (1992). Prevalence of Age-related Maculopathy. The Beaver Dam Eye Study. Ophthalmology.

[3] Nirmalan PK, Robin AL, Katz J, Tielsch JM, Thulasiraj RD, Krishnadas R (2004). Prevalence of vitreoretinal disorders in a rural population of southern India: the Aravind Comprehensive Eye Study. Arch Ophthalmol.

[4] Thapa SS, Thapa R, Paudyal I, Khanal S, Aujla J, Paudyal G (2013). Prevalence and pattern of vitreo-retinal diseases in Nepal: The Bhaktapur glaucoma study. BMC Ophthalmol.

[5] Gupta SK, Murthy GV, Morrison N, Price GM, Dherani M, John N (2007). Prevalence of early and late age-related macular degeneration in a rural population in northern India: the INDEYE feasibility study. Invest Ophthalmol Vis Sci.

[6] Wild S, Roglic G, Green A, Sicree R, King H (2004). Global prevalence of diabetes: estimates for the year 2000 and projections for 2030. Diabetes Care.

[7] World Health Organization. Prevention of blindness from diabetes mellitus: report of a WHO consultation in Geneva, Switzerland, 9–11 November 2005. Geneva 2006.

[8] Woo JH, Sanjay S, AuEong KG (2009). The epidemiology of age-related macular degeneration in the Indian subcontinent. Acta Ophthalmol.

[9] Sapkota YD, Pokharel GP, Nirmalan PK, Dulal S, Maharjan IM, Prakash K (2006). Prevalence of Blindness and cataract surgery in Gandaki Zone, Nepal. Br J Ophthalmol.

[10] Photocoagulation treatment of proliferative diabetic retinopathy. Clinical application of Diabetic Retinopathy Study (DRS) findings, DRS Report Number 8. The Diabetic Retinopathy Study Research Group. Ophthalmology. 1981; 88(7):583–600 7196564

[11] Dandona R, Dandona L, John RK, McCarty CA, Rao GN (2001). Awareness of eye diseases in an urban population in southern India. Bull World Health Organ.

[12] Thapa R, Poudyal G, Maharjan N, Bernstein PS (2012). Demographics and awareness of diabetic retinopathy among diabetic patients attending the vitreo-retinal service at a tertiary eye care center in Nepal. Nepal J Ophthalmol.

[13] Paudyal G, Shrestha MK, Meyer JJ, Thapa R, Gurung R, Ruit S (2008). Prevalence of diabetic retinopathy following a community screening for diabetes. Nepal Med Coll J.

[14] Thapa SS, Rana PP, Twyana SN, Shrestha MK, Paudyal I, Paudyal G (2011). Rationale, methods and baseline demographics of the Bhaktapur Glaucoma Study. Clin Exp Ophthalmol.

[15] International ARM Epidemiological Study Group (1995). An international classification and grading system for age-related maculopathy and agerelated macular degeneration. Surv Ophthalmol.

[16] Brilliant LB, Pokhrel RP, Grasset NC, Lepkowski JM, Kolstad A, Hawks W (1985). Epidemiology of blindness in Nepal. Bull World Health Organ.

[17] Thapa SS, Khanal S, Paudyal I, Twyana SN, Ruit S, Van Rens GHMB (2011). Outcome of cataract surgery: a population based developing world study in the Bhaktapur district, Nepal. Clin Exp Ophthalmol.

[18] Rani PK, Raman R, Subramani S, Perumal G, Kumaramanickavel G, Sharma T (2008). Knowledge of diabetes and diabetic retinopathy among rural populations in India, and the influence of knowledge of diabetic retinopathy on attitude and practice. Rural Remote Health.

[19] Mohan D, Raj D, Shanthirani CS, Datta M, Unwin NC, Kapur A (2005). Awareness and knowledge of diabetes in Chennai--the Chennai Urban Rural Epidemiology Study [CURES-9]. J Assoc Physicians India.

[20] Kadri R (2011). Awareness of diabetic and hypertensive eye disease in public. Int J Biol Med Res.

[21] Thapa R, Joshi DM, Rizyal A, Maharjan N, Joshi RD (2014). Prevalence, risk factors and awareness of diabetic retinopathy among admitted diabetic patients at a tertiary level hospital in Kathmandu. Nepal J Ophthalmol.

[22] Balla SA, Ahmed HA, Awadelkareem MA (2004). Prevalence of diabetes, knowledge and attitude of rural population towards diabetes and hypoglycaemic events, Sudan 2013. Am J Health Res.

[23] Mwangi MW, Githinji GG, Githinji FW (2011). Knowledge and Awareness of Diabetic Retinopathy amongst Diabetic Patients in Kenyatta National Hospital, Kenya. Int J Humanit Soc Sci.

[24] Addoor KR, Bhandary SV, Khanna R, Rao LG, Lingam KD, V SB (2011). Assessment of awareness of diabetic retinopathy among the diabetics attending the peripheral diabetic clinics in melaka, malaysia. Med J Malaysia.

[25] Loewenstein A (2007). The significance of early detection of age-related macular degeneration. Richard and Hinda Rosenthal Foundation Lecture, The Macula Society 29^th^ Annual Meeting. Retina.

[26] Attebo K, Mitchell P, Cumming R, Smith W (1997). Knowledge and beliefs about common eye diseases. Aust NZJ Ophthalmol.

[27] Lau JTF, Lee V, Fan D, Lau M, Michon J (2002). Knowledge about cataract, glaucoma, and age related macular degeneration in the Hong Kong Chinese population. Br J Ophthalmol.

[28] Livingston PM, McCarty CA, Taylor HR (1998). Knowledge, attitudes, and self care practices associated with age related eye disease in Australia. Br J Ophthalmol.

[29] Ng WT, Goggin M (2006). Awareness of and compliance with recommended dietary supplement among age-related macular degeneration patients. Clin Experiment Ophthalmol.

[30] Charkoudian LD, Gower EW, Solomon SD, Schachat AP, Bressler NM, Bressler SB (2008). Vitamin usage patterns in the prevention of advanced age-related macular degeneration. Ophthalmology.

[31] Stevens R, Bartlett H, Walsh R, Cooke R (2014). Age related macular degeneration patient’s awareness of nutritional factors. Br J Vis Impair.

[32] Cimarolli VR, Laben-Baker A, Hamilton WS, Stuen C (2012). Awareness, knowledge, and concern about age-related macular degeneration. Educ Gerontol.

[33] Thapa R, Paudyal G, Shrestha MK, Gurung R, Ruit S (2011). Age-related macular degeneration in Nepal. Kathmandu Univ Med J (KUMJ).

[34] Lawrenson JG, Evans JR (2013). Advice about diet and smoking for people with or at risk of age-related macular degeneration: a cross-sectional survey of eye care professionals in the UK. BMC Public Health.

[35] Chakravarthy U, Augood C, Bentham GC, de Jong PT, Rahu M, Seland J (2007). Cigarette smoking and age-related macular degeneration in the EUREYE Study. Ophthalmology.

[36] Tan JS, Wang JJ, Flood V, Rochtchina F, Smith W, Mitchell P (2008). Dietary antioxidants and the long-term incidence of age-related macular degeneration: the Blue Mountain Eye Study. Ophthalmology.

[37] Thornton J, Edwards R, Mitchell P, Harrison RA, Buchan I, Kelly SP (2005). Smoking and age-related macular degeneration: a review of association. Eye.

[38] Gibson DM (2012). Diabetic retinopathy and age-related macular degeneration in the U.S. Am J Prev Med.

[39] Amos AF, McCarty DJ, Zimmet P (1997). The rising global burden of diabetes and its complications: estimates and projections to the year 2010. Diabet Med.

